# Enhancing Geriatric Knowledge for Interprofessional Health Sciences Students and Skilled Nursing Facility Staff

**DOI:** 10.1093/geroni/igaf122.1357

**Published:** 2025-12-31

**Authors:** Vania Leung

**Affiliations:** University of Illinois Chicago, Chicago, Illinois, United States

## Abstract

My GACA was designed to provide clinical geriatrics and skilled nursing facility training for an interprofessional group of learners to address the need to improve geriatrics care for the skilled nursing facility population. The primary goal of this curriculum is to enhance bidirectional geriatric training and curriculum for health sciences training programs (medicine and nursing) and nursing home staff. The aim is to educate on holistic geriatrics concepts including social determinants of health and disparities in aging, the basic tenets of geriatric clinical medicine and the importance of age-friendly health systems frameworks. This novel, interprofessional approach achieves multiple aims: 1) bridge the gap between what is taught in the classroom and the clinical setting for students and trainees; 2) offer opportunities for existing nursing home staff to better understand and apply core geriatrics knowledge; and 3) provide direct exposure to possible career opportunities for trainees. This presentation will focus on the impact thus far of this program for trainees as well as skilled nursing facility staff and discuss the challenges of implementation. It further discusses the translational aspect of this program on a systemic level across a healthcare system.

